# Long-chain fatty acids as functional modulators in PCOS: metabolic crosstalk, signaling pathways, and therapeutic implications

**DOI:** 10.3389/fnut.2026.1797101

**Published:** 2026-07-01

**Authors:** Cong Wang, Juanjuan Yu, Junyu Zhai, Yifan Wu, Yujiao Wang, Zi-Jiang Chen, Yanzhi Du

**Affiliations:** 1 Department of Reproductive Medicine, Ren Ji Hospital, Shanghai Jiao Tong University School of Medicine, Shanghai, China; 2 Shanghai Key Laboratory for Assisted Reproduction and Reproductive Genetics, Shanghai, China; 3 State Key Laboratory of Reproductive Medicine and Offspring Health, Center for Reproductive Medicine, Institute of Women, Children and Reproductive Health, Shandong University, Jinan, Shandong, China; 4 National Research Center for Assisted Reproductive Technology and Reproductive Genetics, Shandong University, Jinan, Shandong, China

**Keywords:** fatty acid-binding protein, long-chain fatty acids, monounsaturated fatty acids, polycystic ovary syndrome, polyunsaturated fatty acids, saturated fatty acids

## Abstract

Polycystic ovary syndrome (PCOS), a prevalent endocrine-metabolic disorder, exhibits dysregulated lipid metabolism with distinct alterations in long-chain fatty acids (LCFAs) profile. Heterogeneous profile of LCFAs dysregulation manifest in distinct PCOS phenotypes: Saturated fatty acids (SFAs) preferentially accumulate in serum and follicular fluid (FF) of insulin-resistant/hyperandrogenic (IR/HA) subtypes, exacerbating lipotoxicity, inflammatory responses, and ovarian impairment; monounsaturated fatty acids (MUFAs) display compensatory elevation yet demonstrate context-dependent effects regulated by stearoyl-CoA desaturase-1 (SCD1) activity; and polyunsaturated fatty acids (PUFAs) exhibit subtype-dependent actions: n-3 derivatives (EPA/DHA) ameliorate metabolic dysfunction, resolve inflammation, and enhance ovarian competence, whereas pro-inflammatory n-6 PUFAs elevate the n-6/n-3 ratio, disrupting follicular homeostasis. In PCOS, dysregulation of LCFA transport receptors, including fatty acid-binding proteins 4/5 (FABP4/5), CD36, are associated with insulin resistance (IR) and hyperandrogenemia (HA) through obesity-independent mechanisms. Sensing receptors GPR120 and GPR40 critically orchestrate insulin sensitivity, inflammatory cascades, and reproductive function, highlighting their therapeutic relevance in PCOS pathophysiology. Although direct evidence remains limited, targeting these pathways may represent a promising strategy to concurrently mitigate HA, IR, and dyslipidemia in a phenotype-specific manner, warranting further preclinical and clinical investigations. Future investigations should focus on defining and validating clinical biomarkers, tissue-specific receptor functions, and evaluating targeted interventions in distinct PCOS phenotypes.

## 1 Introduction

Polycystic ovary syndrome (PCOS) is a widespread endocrine disorder impacting roughly one in eight women of reproductive age globally. The diagnostic criteria for PCOS remain inconsistent, and the internationally recognized standard continues to be the Rotterdam criteria (1). According to the Rotterdam criteria, a patient must exhibit at least two of the following three criteria, while excluding other potential diseases, to receive a PCOS diagnosis: (i) OD (oligomenorrhea or anovulation), (ii) HA (hyperandrogenism), and (iii) PCOM (polycystic ovary morphology). These criteria delineate four distinct phenotypes: phenotype A (OD + HA + PCOM), phenotype B (OD + HA), phenotype C (HA + PCOM), and phenotype D (OD + PCOM). The clinical presentation of PCOS is remarkably heterogeneous, manifesting across several phenotypes that vary significantly by life stage, race and/or ethnicity, and degree of adiposity. Matthew et al. employed an unsupervised clustering method, revealing three distinct subgroups of women with PCOS characterized by different underlying genetic risk factors: a “reproductive” group marked by elevated LH (luteinizing hormone) and SHBG (sex hormone-binding globulin) levels, alongside relatively low BMI (Body mass index) and insulin; a “metabolic” group defined by significantly elevated BMI, glucose, and insulin levels; and an “indeterminate” group, presenting with lower LH and SHBG levels (2). The etiology of PCOS remains elusive, yet compelling evidence indicates it is a polygenic disorder intricately woven with epigenetic, developmental, and environmental factors. Growing evidence strongly supports defining PCOS as a profoundly heterogeneous metabolic syndrome. Crucially, insulin resistance and metabolic dysfunction play a pivotal role in the syndrome’s pathogenesis, driving numerous adverse metabolic outcomes that impose a significant burden on the lifelong health of women with PCOS (3). Furthermore, mounting evidence increasingly demonstrates that obesity significantly impairs folliculogenesis, oogenesis, embryo development, and implantation. Beyond its well-established connection to anovulatory infertility, PCOS substantially heightens the risk of metabolic syndrome-characterized by obesity, insulin resistance (IR), and dyslipidemia (4). These effects occur through direct mechanisms or indirect pathways mediated by metabolic, immune, and secretory systems (5). Moreover, recent studies suggest that PCOS is independently associated with chronic inflammation, even when metabolic confounders are excluded (6). Crucially, patients exhibit elevated low-density lipoprotein cholesterol (LDL-C) and non-high-density lipoprotein cholesterol (non-HDL-C) levels even independent of body mass index (BMI), strongly suggesting obesity-independent dyslipidemic pathways (7). Stepwise Mendelian randomization analyses pinpoint elevated BMI and reduced HDL-C as primary genetic drivers of PCOS pathogenesis, underscoring their central role in disease etiology (8). The intricate pathophysiological interplay among obesity, IR, and HA exerts both independent and synergistic effects on lipid metabolism. The studies cited herein utilize different classification systems to delineate PCOS heterogeneity, which necessitates clarification for cross-comparison. The internationally recognized Rotterdam criteria define phenotypes based on clinical signs: phenotype A (OD + HA + PCOM), phenotype B (OD + HA), phenotype C (HA + PCOM), and phenotype D (OD + PCOM). Concurrently, data-driven cluster analyses have identified subgroups such as the “metabolic” subtype (characterized by prominent insulin resistance, dyslipidemia, and higher BMI) and the “reproductive” subtype (marked by elevated LH with less severe metabolic disturbances). Importantly, there is partial overlap between these systems. The “metabolic PCOS” cluster partially overlap with individuals who would be classified under Rotterdam phenotypes A and C, and is shares important metabolic features with the cohort described in many studies as “IR-PCOS.” Similarly, the “reproductive” cluster shows overlap with phenotypes B and D. In the following sections, when citing findings based on a specific classification (e.g., “IR-PCOS,” “metabolic subtype”), we will note its likely correspondence to the other system to facilitate a unified interpretation of the data.

Mounting evidence positions long-chain fatty acids (LCFAs; C13-22)-dominant components of dietary fats and adipose reserves-as pivotal molecular hubs integrating profound metabolic dysregulation with PCOS pathology (9). LCFAs fulfill multifaceted roles: structurally integrating into phospholipid bilayers to modulate membrane fluidity and receptor organization; energetically fueling *β*-oxidation while generating prostaglandins to regulate follicular angiogenesis; and orchestrating intricate signaling via protein lipidation and membrane receptor activation (e.g., GPR120, GPR40), directly governing insulin sensitivity, steroidogenesis, and inflammatory cascades (10). The homeostatic balance of LCFAs is critical for maintaining reproductive and metabolic health, and its disruption is increasingly recognized as a key driver of pathophysiological processes in ovarian disorders. PCOS patients often display a dyslipidemic profile with elevated levels of saturated LCFAs and reduced proportions of unsaturated LCFAs. These alterations not only exacerbate systemic IR via the toll-like receptor 4 (TLR4)/nuclear factor-κB (NF-κB) inflammatory axis but also directly impair ovarian function. Despite growing evidence linking LCFAs dysmetabolism to PCOS pathogenesis, the precise molecular mechanisms by which specific LCFAs modulate the crosstalk between ovarian somatic cells (granulosa, theca, and stromal cells) and immune cells (macrophages, T cells) within the follicular microenvironment remain understudied. Furthermore, the potential of targeting LCFAs metabolism-via dietary intervention or pharmacological modulation of lipid sensors like GPR120-as a therapeutic strategy for PCOS-associated reproductive and metabolic abnormalities warrants further investigation (11). The high heterogeneity inherent in PCOS, combined with the current limitations of clinical diagnostic methods, presents formidable challenges for achieving truly precise treatment strategies. By analyzing how the disease profoundly impairs LCFA metabolic pathways within the body, this approach facilitates precise diagnosis and targeted intervention at the molecular level, laying the groundwork for a decisive transition to precision medicine in PCOS management.

## 2 Classification, nomenclature and metabolism of LCFAs

### 2.1 Structural taxonomy of fatty acids: chain length, unsaturation, and nomenclature

Fatty acids (FAs) are primarily classified by carbon chain length into: short-chain (SCFAs, 2-6C), medium-chain (MCFAs, 7–12C), long-chain (LCFAs, 13-22C), and very long-chain (VLCFAs, >22C) fatty acids (12). LCFAs are further categorized by saturation status: saturated (SFAs, no double bonds), monounsaturated (MUFAs, one double bond), and polyunsaturated (PUFAs, ≧2 double bonds). PUFAs are subclassified via *ω*-positioning of the first double bond from the methyl terminus into ω-3, ω-6, or ω-9 families, with geometric isomerism (cis/trans) defining conformational subtypes. In humans, FAs predominantly exhibit even-numbered chains (C14-C24), with C16 and C18 being most abundant.

**Figure 1:**
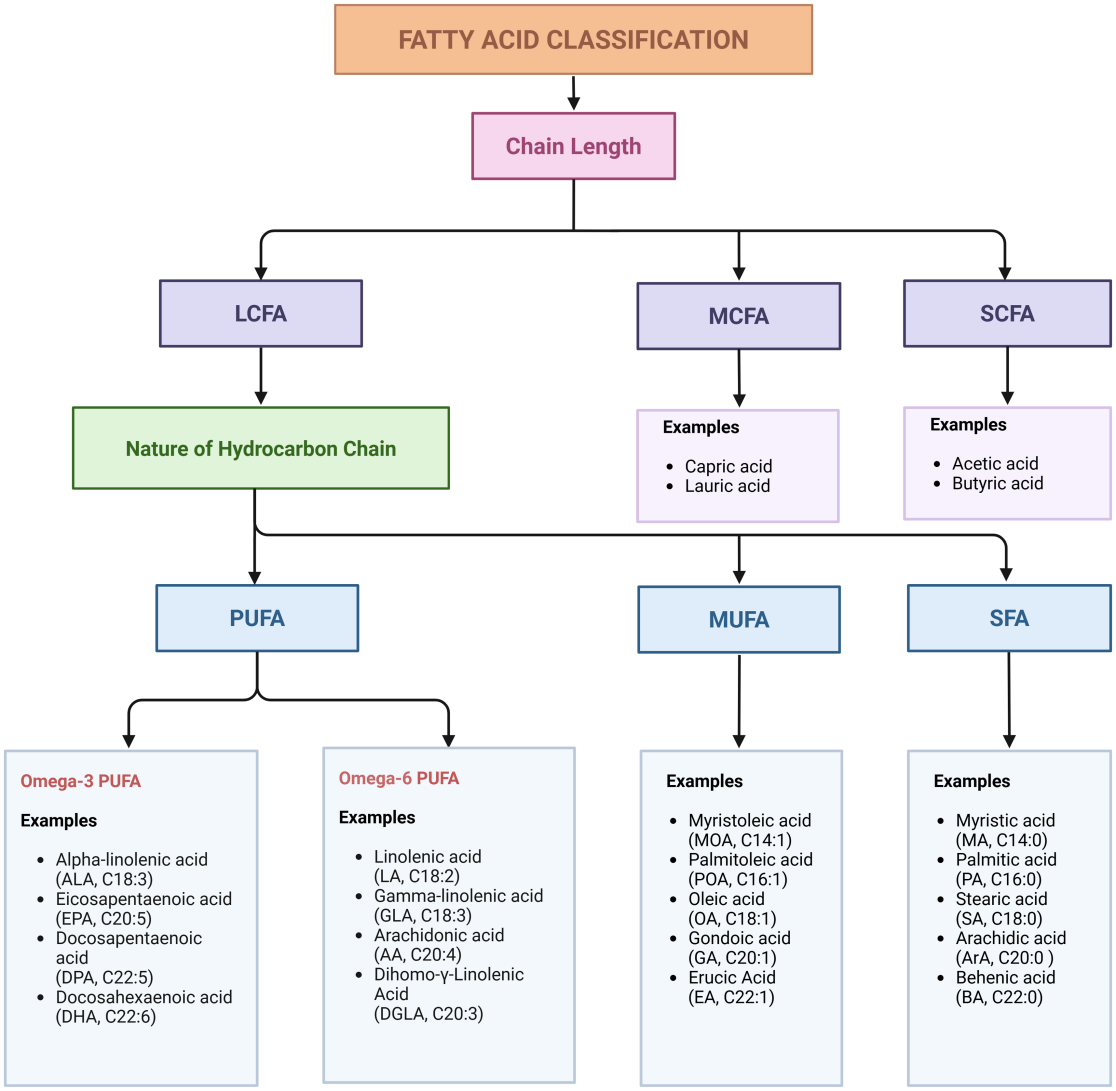
Systematic classification of fatty acids: chain length and structural diversity (Created with BioRender.com, agreement number: RJ29VKDINV). This hierarchical flowchart illustrates the systematic classification of fatty acids according to two primary criteria: chain length and hydrocarbon chain structure. Fatty acids are initially categorized by carbon chain length into long-chain fatty acids (LCFA, >12 carbons), medium-chain fatty acids (MCFA, 7–12 carbons), and short-chain fatty acids (SCFA, ≤6 carbons). MCFA and SCFA are further exemplified with representative species: capric acid (C10:0) and lauric acid (C12:0) for MCFA; acetic acid (C2:0) and butyric acid (C4:0) for SCFA. LCFA are subsequently classified based on the nature of their hydrocarbon chain into polyunsaturated fatty acids (PUFA), monounsaturated fatty acids (MUFA), and saturated fatty acids (SFA). PUFA are subdivided into omega-3 and omega-6 families based on the position of the first double bond from the methyl end. Omega-3 PUFA include alpha-linolenic acid (ALA, C18:3), eicosapentaenoic acid (EPA, C20:5), docosapentaenoic acid (DPA, C22:5), and docosahexaenoic acid (DHA, C22:6). Omega-6 PUFA include linoleic acid (LA, C18:2), gamma-linolenic acid (GLA, C18:3), arachidonic acid (AA, C20:4), and dihomo-*γ*-linolenic acid (DGLA, C20:3). MUFA examples include myristoleic acid (C14:1), palmitoleic acid (C16:1), oleic acid (C18:1), gondoic acid (C20:1), and erucic acid (C22:1). SFA examples include myristic acid (C14:0), palmitic acid (C16:0), stearic acid (C18:0), arachidic acid (C20:0), and behenic acid (C22:0). This classification framework provides a comprehensive overview of fatty acid diversity and serves as a foundation for understanding their metabolic roles, dietary sources, and biological functions in health and disease.

The naming of LCFAs systematically reflects their structural features: double bond positions are numbered from the carboxyl group (*Δ*-numbering), as exemplified by arachidonic acid (AA), formally designated (5Z, 8Z, 11Z, 14Z)-eicosa-5,8,11,14-tetraenoic acid. A simplified delta notation (C20: 4n-6) concisely denotes chain length (20 carbons), double bond count (4), and their omega/n- classification (first bond at the sixth carbon from the methyl terminus) (13). The common classification and nomenclature of LCFAs are presented in Figure 1.

### 2.2 Enzymatic regulation of LCFAs’ metabolism: ACC/FASN-driven synthesis and desaturase-mediated modification

LCFAs are primarily obtained through dietary intake, though endogenous biosynthesis occurs via enzymatic cascades initiating with acetyl-CoA carboxylase (ACC) converting acetyl-CoA to malonyl-CoA. Fatty acid synthase (FASN) then catalyzes malonyl-CoA condensation to generate palmitic acid (C16: 0), which undergoes elongation by Elovl enzymes and desaturation by stearoyl-CoA desaturases (SCDs) or fatty acid desaturases (FADSs) to produce mono- and polyunsaturated fatty acids (MUFAs/PUFAs), conferring metabolic flexibility (14). While most LCFAs can be synthesized endogenously, essential fatty acids—including *α*-linolenic acid (ALA, C18:3n-3) and linoleic acid (LA, C18:2n-6)—require dietary supplementation as precursors for n-3/n-6 PUFA synthesis due to limited mammalian conversion efficiency. Emerging evidence suggests gut microbiota modulate LCFA bioavailability, though mechanistic links to pathologies like PCOS remain unclear (15, 16).

LCFAs act as ligands for peroxisome proliferator-activated receptors (PPARs), which regulate energy metabolism and help maintain their metabolic activity and insulin sensitivity (17). Dysfunction in fatty acid oxidation (FAO), steroid metabolism, and their interactions may contribute to the pathogenesis of PCOS in the ovary. However, there is limited knowledge regarding whether the oxidative catabolism of LCFA is abnormal in PCOS patients. Additionally, apart from serving as a source of oxidative energy and participating in plasma membrane lipid composition, LCFAs can also generate bioactive derivatives that play a role in regulating physiological functions-especially PUFAs.

This review comprehensively analyzes how LCFAs and their cognate receptors-spanning transporters (CD36, FABP4/5) and signaling receptors (FFAR1/4, also known as GPR40/120)-precisely coordinate these interconnected axes to propel PCOS development, with a translational focus on their therapeutic promise for concurrently ameliorating hyperandrogenism, insulin resistance, and lipid dysregulation in a phenotype-specific manner.

## 3 LCFA subtypes in PCOS: metabolic dysregulation and phenotypic paradoxes

Although there are numerous studies investigating the roles and mechanisms of LCFAs in the occurrence, development, and treatment of PCOS, most consist of clinical studies with limited mechanistic depth. PCOS is a highly heterogeneous condition, and the research findings show inconsistencies. This review summarizes current evidence related to LCFAs and PCOS from the three major subtypes of LCFAs (SFAs, MUFAs, PUFAs).

### 3.1 SFAs in PCOS: phenotype-specific metabolic and reproductive dysregulation

**Figure 2:**
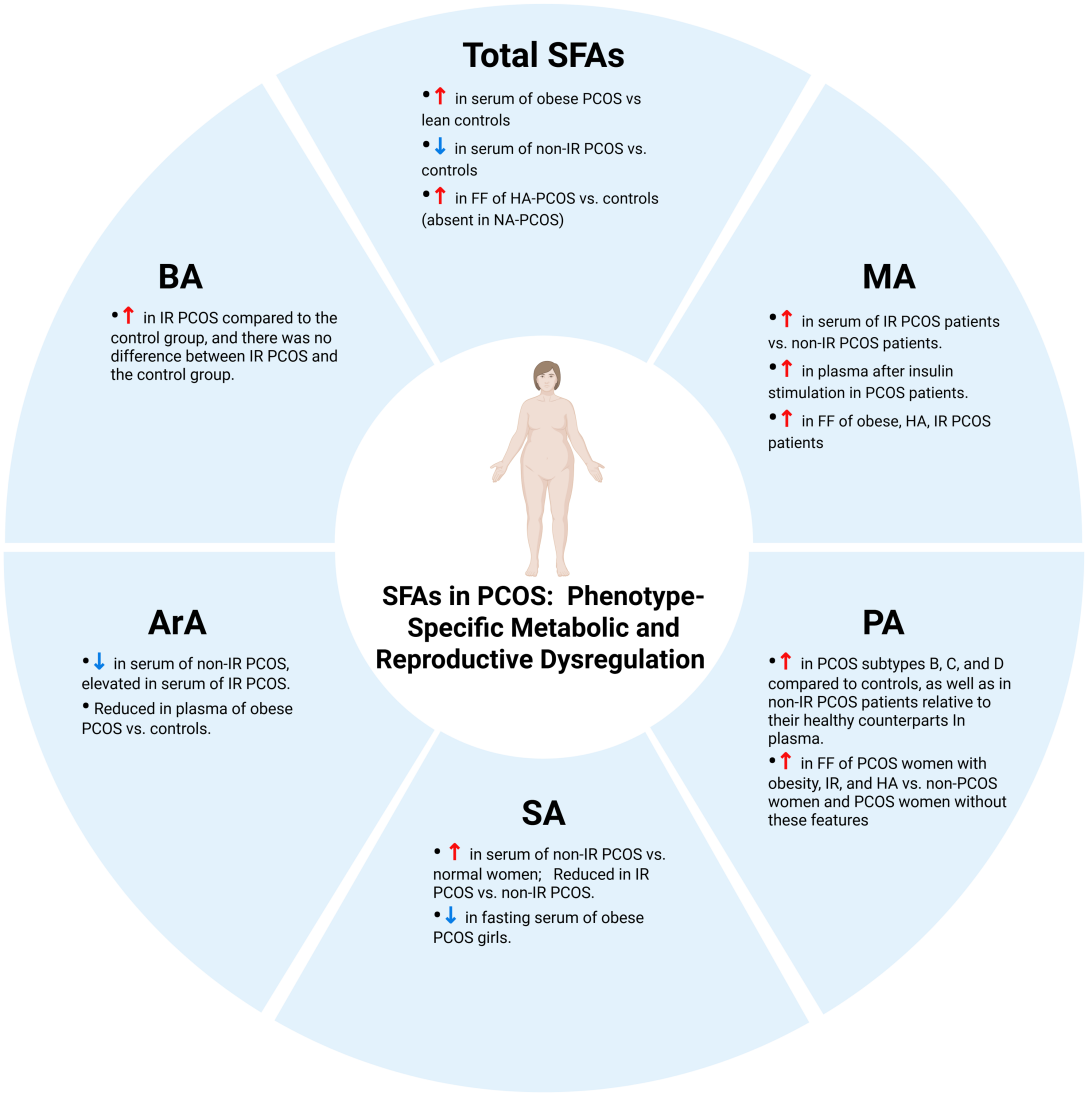
Saturated fatty acids in PCOS: phenotype-specific metabolic and reproductive dysregulation (Created with BioRender.com, agreement number: WB29VKF2W8). This circular schematic illustrates the altered profiles of major saturated fatty acids (SFAs)-myristic acid (MA, C14:0), palmitic acid (PA, C16:0), stearic acid (SA, C18:0), arachidic acid (ArA, C20:0), and behenic acid (BA, C22:0)-in polycystic ovary syndrome (PCOS) across distinct metabolic and phenotypic subgroups. Central to the diagram is the concept that SFA dysregulation contributes to both metabolic disturbances and reproductive dysfunction in PCOS, with patterns varying significantly by phenotype. Key findings include: Total SFAs are elevated in serum of obese PCOS patients compared to lean controls but reduced in non-insulin-resistant (non-IR) PCOS versus healthy controls; they are also increased in follicular fluid (FF) of hyperandrogenic (HA)-PCOS women relative to controls, a change not observed in non-HA PCOS. Myristic acid (MA) is elevated in serum of insulin-resistant (IR) PCOS patients compared to non-IR PCOS and increases post-insulin stimulation in PCOS, suggesting dynamic metabolic involvement; it is also elevated in FF of obese, HA, IR PCOS. Palmitic acid (PA) is increased in PCOS subtypes B, C, and D, as well as in non-IR PCOS compared to healthy controls, and is further elevated in FF of PCOS women with obesity, IR, and HA versus those without these features. Stearic acid (SA) is increased in serum of non-IR PCOS vs. normal women but decreased in IR PCOS vs. non-IR PCOS and reduced in fasting serum of obese PCOS girls. Arachidic acid (ArA) is decreased in serum of non-IR PCOS but elevated in IR PCOS and reduced in plasma of obese PCOS versus controls. Behenic acid (BA) is elevated in IR PCOS compared to controls, with no difference between IR PCOS and controls in other comparisons. These data highlight that SFA alterations are not uniform across PCOS phenotypes and reflect underlying metabolic heterogeneity, implicating specific SFAs as potential biomarkers or mediators of PCOS pathophysiology.

SFAs are the most abundant LCFAs in the body. Their primary role is to contribute to plasma membrane composition and serve as an oxidative energy source. Additionally, SFAs serve as substrates for protein modifications, such as palmitoylation and myristoylation, which can alter protein function. Serum SFA levels in obese PCOS patients were found to be significantly elevated compared to those in lean controls and lean PCOS patients (18, 19). Even after adjusting for BMI, women with PCOS exhibited significantly higher serum total unsaturated fatty acid levels compared to healthy controls (20). Non-IR PCOS patients showed significantly lower total SFA levels compared to controls, whereas IR-PCOS patients show a significant increase in total SFAs relative to non-IR PCOS subjects (21). In HA-PCOS women, a dramatic elevation in SFA levels in follicular fluid (FF) was noted, an alteration absent in non-HA PCOS cases (22). The increase in total SFA levels in women with PCOS may be attributed to higher dietary intake of SFAs, with a positive correlation between SFA consumption and PCOS risk. Moreover, reduced SFA intake has been associated with improved cardiovascular autonomic function in PCOS patients (23). Although total SFA levels did not differ significantly between the reproductive and metabolic subtypes of PCOS (20), distinct alterations in individual SFAs-such as elevated myristic acid (MA, C14: 0) in insulin-resistant PCOS, increased palmitic acid (PA, C16:0) in phenotypes B, C, and D, and phenotype-specific changes in stearic acid (SA, C18:0)-have been linked to key metabolic and reproductive features, including IR, HA, and obesity. The research findings on specific SFA subtypes in PCOS are summarized in Figure 2 and Table 1, and are discussed in detail in Section 3.1.1–3.1.3.

**Table 1:** Summary of the association between SFA and PCOS.

LCFA	Research Type	Sample	Conclusion	Ref
Total SFA	Observational	Serum	There was no significant change in the total SFA of PCOS, but the SFA% reduce. Compared with the overweight control group, the SFA% of overweight PCOS group decrease. Compared with the lean PCOS, the total SFA of the overweight PCOS group increased.	(18)
Total SFA	Observational	Serum	Obese patients with PCOS had increased levels of long-chain saturated fatty acids vs. lean controls	(19)
Total SFA	Observational	Serum	Total SFA levels were significantly lower in non-IR PCOS	(21)
Total SFA	Observational	Serum	Total SFA levels in serum of PCOS reproductive subtypes are not significantly different from metabolic subtypes.	(20)
Total SFA	Observational	FF	Compared with the controls, SFAs in FFs of HA-PCOS women were dramatically increased, while this alteration was not visible in the NA-PCOS group.	(22)
MA	Observational	Serum	MA was also significantly increased in IR PCOS patients compared with non-IR PCOS patients	(24)
MA	Observational	Serum	MA was significantly associated with 2 h post-OGTT glucose, fasting insulin, and HOMA-IR.	(20)
MA	Observational	Plasma	MA significantly increased after insulin stimulation in PCOS.	(25)
MA	Observational	FF	MA is elevated in obese, HA, IR PCOS patients.	(26)
PA	Observational	FF	The concentration of PA of PCOS women in the obesity, IR, and HA groups was higher than that in non-PCOS women, and higher than that in PCOS women without obesity, IR, and HA.	(26)
PA	*In vitro*	KGN cell and mouse primary GCs	Glucose uptake in cultured GCs and lactate accumulation were stimulated by insulin, but the effects of insulin were attenuated by PA treatment. Insulin-induced phosphorylation of Akt was reduced by PA in a dose- and time-dependent manner. PA increased phosphorylation of JNK and JNK blockage rescued the phosphorylation of Akt which was downregulated by PA.	(31)
PA	*In vitro*	KGN cell and human primary GCs	PA can induce decreased FSHR expression levels FSHR expression and estradiol (E2) production via the TRIB3/Akt/GSK3βpathway.	(35)
PA	*In vitro*	Human primary GCs	PA increased the enrichment of HIF-1α at the binding site region of the LOX promoter in human granulosa cells, leading to local ECM remodeling dysregulation.	(30)
PA/SA	Observational	Plasma	PCOS types B, C, and D show higher PA content than controls, with no significant difference in type A. Types B and D also have higher SA content compared to controls, while types A and C show no significant difference.	(9)
PA/SA	Observational	Plasma	Non-IR PCOS patients have significantly higher PA and SA levels than normal women; in IR PCOS, SA decreases and PA trends downward compared to non-IR PCOS.	(29)
PA/SA	Observational	Plasma	Obese girls with PCOS have lower fasting SA levels.	(25)
PA/SA	*In vitro*	Human primary GCs	PA and SA, significantly inhibited granulosa cell survival in a time and dose dependent manner. The PUFA, instead shows a protective effect against PA and SA-induced apoptosis.	(32)
Eicosanoic acid	Observational	Serum	Eicosanoic acid decreased in non-IR PCOS, elevated in IR PCOS.	(21)
Eicosanoic acid	Observational	Plasma	Obese women with PCOS have lower fasting eicosanoic acid compared with control.	(25)
BA	Observational	Plasma	BA was increased in IR PCOS compared to the control group, and there was no difference between IR PCOS and the control group.	(21)

PCOS, polycystic ovary syndrome; SFA, saturated fatty acid; MUFA, monounsaturated fatty acid; PUFA, polyunsaturated fatty acid; IR, insulin resistance; FF, follicular fluid; MA, myristic acid; OGTT, oral glucose tolerance test; HOMA-IR, homeostasis model assessment of insulin resistance; PA, palmitic acid; KGN cells, human granulosa-like tumor cell line; GCs, granulosa cells; FSHR, follicle-stimulating hormone receptor; E2, estradiol; HIF-1α, hypoxia-inducible factor 1-alpha; LOX, lysyl oxidase; ECM, extracellular matrix; SA, stearic acid; NMR, nuclear magnetic resonance; BA, behenic acid.

#### 3.1.1 Myristic acid (MA, C14:0): a context-dependent dual actor in PCOS

MA, a 14-carbon SFA, exhibits context-dependent dual roles in PCOS. Clinical studies report significantly elevated MA levels in PCOS patients, particularly in those with IR, where its concentrations are markedly higher compared to non-IR individuals (24). MA shows strong correlations with metabolic dysfunction, including impaired glucose tolerance, elevated fasting insulin, and increased homeostatic model assessment of insulin resistance (HOMA-IR) indices (20). Notably, MA levels further increase following insulin stimulation in PCOS patients, suggesting a feedforward loop between HA and MA accumulation (25). This association extends to the ovarian microenvironment, where free MA levels are disproportionately elevated in the FF of obese, HA, and IR-PCOS patients, implicating localized lipotoxicity in reproductive dysfunction (26). Paradoxically, while MA demonstrates protective effects—it exacerbates adipose tissue inflammation and systemic insulin resistance in obesity mouse models (27). Mechanistically, this duality may arise from MA’s involvement in protein myristoylation, a post-translational modification critical for protecting against diet-induced metabolic disorders and modulating immune responses (28). Currently, it remains unclear whether myristoylation-related proteins may be involved in the pathogenesis of PCOS.

#### 3.1.2 Palmitic acid (PA, C16:0) and stearic acid (SA, C18:0): phenotype-specific dysregulation

PA and SA, the predominant SFAs in humans, exhibit phenotype-specific dysregulation in PCOS. PCOS subtypes B, C, and D display elevated serum PA levels compared to control women, while there is no significant difference in type A. Similarly, serum SA content increases selectively in subtypes B and D, with no alteration observed in subtypes A and C (9). Non-IR PCOS patients exhibit higher circulating PA and SA than healthy women, but SA levels decline in IR-PCOS patients compared to non-IR counterparts, with PA showing a similar downward trend. Obese girls with PCOS exhibit lower fasting SA levels (25, 29). Analogous to the circulation variations, PA levels in FF were higher in PCOS women with obesity, IR, or HA than in non-PCOS women and PCOS counterparts without these conditions (26). Generally speaking, elevated levels of PA and SA were observed in both serum and FF of PCOS patients. Moreover, factors such as obesity, IR, and HA may exacerbate the accumulation of these SFAs, consequently inducing lipotoxicity.

Potential mechanisms may involve PA/SA overload contributing to lipotoxic cascades through toll-like receptors (TLRs)/NF-κB-mediated inflammatory responses, mitochondrial reactive oxygen species (ROS) overproduction, and ER stress-induced autophagy disruption. In ovarian stroma, PA might dysregulate HIF-1α/LOX-driven collagen remodeling, potentially promoting abnormal extracellular matrix (ECM) deposition and anovulation (30). Granulosa cell (GC) studies suggest PA/SA could induce apoptosis via JNK pathway activation (31). JNK pathway activation by PA can also lead to insulin resistance (31), a process that PUFA supplementation appears to mitigate (32). Conversely, similar to MA, PA can act as substrates for protein palmitoylation or myristoylation modifications, thereby regulating metabolism and immune responses (33, 34). Notably, PA has been observed to suppress follicle-stimulating hormone receptor (FSHR) expression and estradiol (E2) production in human ovarian granulosa tumor cell line (KGN) (35). Furthermore, RNA-seq transcriptome analysis revealed that high Level of LCFAs (mainly SFAs) primarily affected the expression of genes related to glucose and insulin homeostasis, fatty acid metabolism, steroidogenesis, and GC differentiation processes (36).

#### 3.1.3 Longer-chain SFAs in PCOS: distinct metabolic profiles

Longer chain SFAs (LC-SFAs) exhibit distinct metabolic profiles in PCOS. Obese women with PCOS demonstrate reduced fasting eicosanoic acid (C20:0) levels compared to controls, with non-IR PCOS subgroups showing further suppression contrasting paradoxical elevations in IR-PCOS patients (21, 25). Behenic acid (BA, C22:0), a representative LC-SFA, displays IR-specific dysregulation: elevated in IR-PCOS relative to non-IR individuals, though no significant difference emerges between IR-PCOS and the control group (21). Despite low bioavailability, BA potentially exacerbates dyslipidemia by elevating cholesterol levels. Circulating BA concentrations are influenced by dietary saturated fat intake, and due to its lower bioavailability than other FAs, BA has been shown to raise cholesterol levels in the human body (37). Intriguingly, while elevated BA associates with reduced cardiovascular mortality in general populations (38), its direct role in PCOS pathogenesis remains undefined, highlighting disease-specific metabolic paradoxes.

#### 3.1.4 SFA-mediated mechanisms linking obesity and PCOS

Growing evidence indicates that SFAs may mediate the interplay between obesity and PCOS. Excessive SFA intake contributes to obesity while dysfunctional white adipose tissue (WAT) in obese patients can exacerbate PCOS. SFAs are traditionally considered to be lipotoxic, and high concentrations of LCFAs (predominantly SFAs) can potentially impair fertility by altering follicular physiology and reducing oocyte developmental capacity. This alteration also affects gene expression related to energy/FAs/steroid metabolism, apoptosis, and OS in GCs (39). Elevated circulating levels of SFAs (such as palmitic and stearic acid) during metabolic stress can significantly hinder oocyte and embryonic development (39, 40). Studies in high-fertility phenotype mice reveal that lower plasma SFAs concentrations and distinct ovarian lipid microenvironments may contribute to enhanced fertility (41). Additionally, excessive follicular SFA exposure suppresses oocyte maturation by inducing intracellular OS, which could partially explain reproductive dysfunction in females with PCOS. Notably, fatty acids exhibit dual roles as essential oocyte/embryo nutrients and potential cytotoxins, necessitating precise regulation of their beneficial versus detrimental effects to optimize developmental outcomes (40). Furthermore, apart from direct interaction between SFA and the ovary contributing to PCOS, there are complex and subtle interactions between SFA-induced neuroendocrine signaling and the hypothalamic–pituitary-ovarian (HPO) axis. Disruptions in this intricate balance can lead to metabolic and reproductive disorders.

### 3.2 MUFAs and PCOS: metabolic dysregulation, compensatory adaptation, and therapeutic paradoxes

#### 3.2.1 SCD1: a metabolic arbiter in MUFAs biosynthesis

MUFAs are LCFAs containing one double bond that can be acquired through dietary intake or endogenous synthesis. Stearoyl-CoA desaturase 1 (SCD1), the Δ9-desaturase converting SFAs to MUFAs, serves as a critical metabolic nexus in PCOS. Nutritional factors tightly regulate SCD1 expression and activity, with SFAs upregulating and PUFAs suppressing its function, establishing a dynamic balance influenced by dietary patterns. Aberrant transcription and epigenetic activation of SCD1 disrupt lipid homeostasis by dysregulating AMP-activated protein kinase (AMPK)/acetyl-CoA carboxylase (ACC), sirtuin 1 (SIRT1)/peroxisome proliferator-activated receptor gamma coactivator 1-alpha (PGC1α) signaling pathways, driving abnormal lipid accumulation. Crucially, SCD1 activity is universally elevated in PCOS patients regardless of obesity status (18). Beyond PCOS-specific pathology, these molecular perturbations contribute to the progression of broader metabolic diseases, including obesity, non-alcoholic fatty liver disease, and diabetes (42).

**Figure 3:**
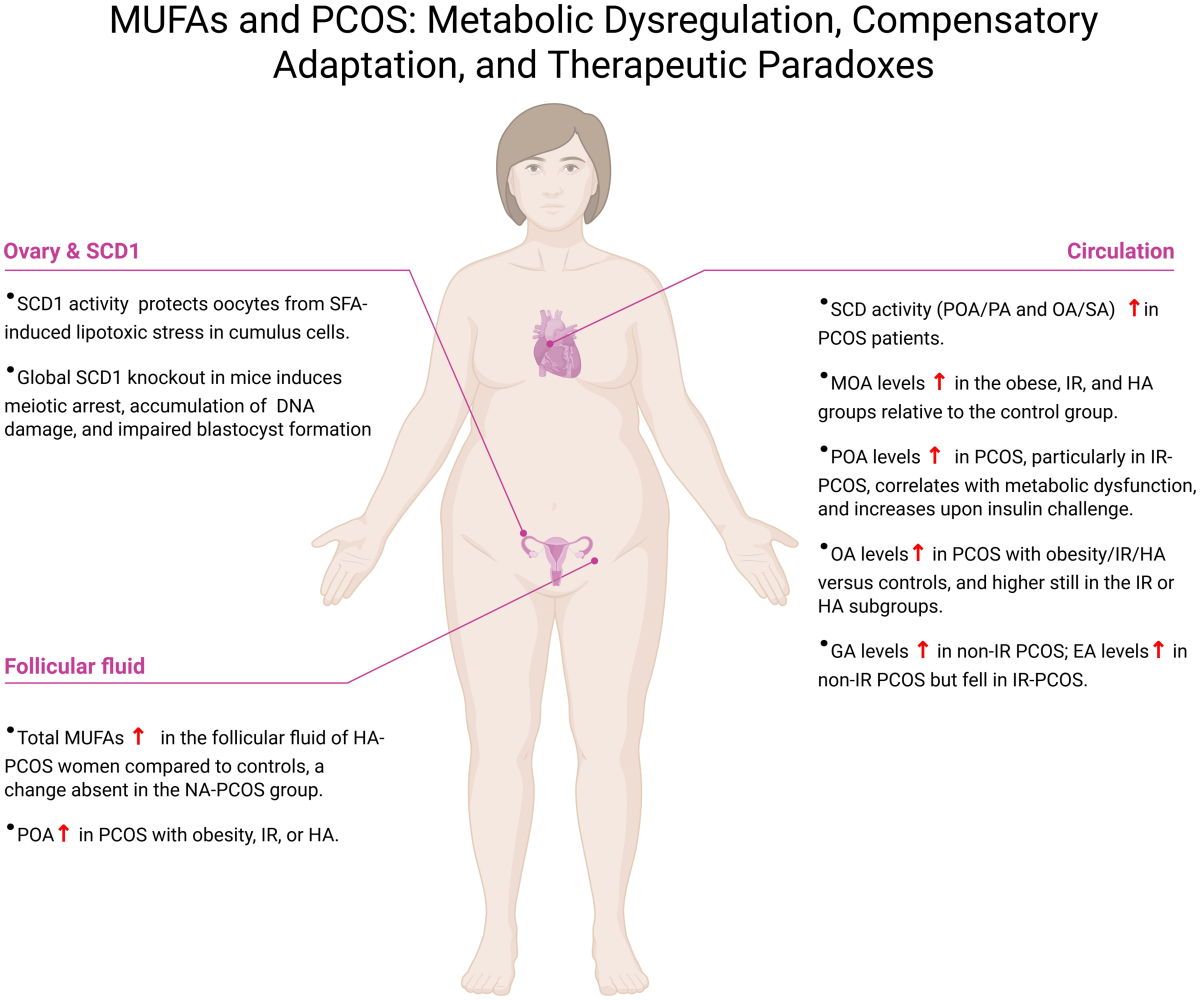
Monounsaturated fatty acids and PCOS: metabolic dysregulation, compensatory adaptation, and therapeutic paradoxes (Created with BioRender.com, agreement number: KQ29VKF4D5). This schematic illustrates the dysregulation of monounsaturated fatty acids (MUFAs) and stearoyl-CoA desaturase 1 (SCD1) activity in polycystic ovary syndrome (PCOS), highlighting tissue-specific alterations in ovarian, follicular fluid, and systemic compartments. In the ovary, SCD1 activity protects oocytes from saturated fatty acid (SFA)-induced lipotoxic stress in cumulus cells; global SCD1 deficiency in mice leads to meiotic arrest, DNA damage accumulation, and impaired blastocyst formation, underscoring its essential role in reproductive health. In follicular fluid, total MUFAs are elevated in hyperandrogenic (HA)-PCOS women compared to controls, a change not observed in non-hyperandrogenic (NA)-PCOS, suggesting a compensatory response to lipid overload in HA-PCOS. Palmitoleic acid (POA) is increased in PCOS with obesity, insulin resistance (IR), or hyperandrogenism (HA). In circulation, SCD activity (reflected by POA/PA and oleic acid/stearic acid ratios) is elevated in PCOS patients, indicating enhanced desaturation as a metabolic adaptation. Myristoleic acid (MOA) levels rise in obese, IR, and HA subgroups relative to controls. POA levels are increased in PCOS, particularly in IR-PCOS, and correlate with metabolic dysfunction, further increasing after insulin challenge. Oleic acid (OA) levels are elevated in PCOS with obesity/IR/HA versus controls, with even higher levels in IR or HA subgroups. Gondoic acid (GA) increases in non-IR PCOS, while erucic acid (EA) rises in non-IR PCOS but declines in IR-PCOS. These findings reveal a complex interplay between MUFA metabolism, insulin resistance, and reproductive pathology in PCOS, where compensatory upregulation of SCD1 may paradoxically contribute to metabolic inflexibility and ovarian dysfunction, posing challenges for therapeutic targeting.

Elevated SCD activity, as reflected by increased product/precursor ratios (e.g., POA/PA), has been observed in the serum of PCOS patients (18). Furthermore, total MUFAs, products of SCD1, are elevated in the follicular fluid of HA-PCOS women (22). This widespread dysregulation of SCD1 pathway activity across multiple compartments underscores its complex metabolic role in PCOS. Interestingly, SCD1 activity in cumulus cells has been demonstrated to protect oocytes from SFA-induced lipotoxic stress (43). Conversely, global SCD1 knockout in mice induces meiotic arrest, accumulation of DNA damage, and impaired blastocyst formation (44), highlighting its indispensable role in reproductive competence. While some studies report that SCD1 inhibition exacerbates SFA-induced lipotoxicity, others demonstrate beneficial metabolic outcomes (45). This functional paradox warrants further investigation into SCD1’s precise role in inflammatory and PCOS pathogenesis. Research findings on specific MUFA subtypes in PCOS are summarized in Figure 3 and Table 2.

**Table 2:** Summary of the association between MUFA and PCOS.

LCFA	Research Type	Sample	Conclusion	Ref
Total MUFA	Observational	FF	Compared with the controls, MUFAs in FFs of HA-PCOS women were dramatically increased, while this alteration was not visible in the NA-PCOS group.	(22)
POA/PA OA/SA	Observational	Serum	Regardless of being lean or overweight, SCD activity (POA/PA and OA/SA) increased in PCOS patients.	(18)
MOA	Observational	FF	The MOA content of the obese group, IR group and HA group was higher than that of the control group	(26)
POA	Observational	Serum	POA increase in PCOS serum, IR-PCOS has higher levels of POA compared with NIR-PCOS.	(24)
POA	Observational	Serum	POA was significantly associated with 2 h post-OGTT glucose, fasting insulin, HOMA-IR, and leptin levels.	(20)
POA	Observational	Plasma	POA significantly increased after insulin stimulation in PCOS.	(25)
POA	Observational	FF	POA concentrations were higher in PCOS subgroups with obesity, IR, or HA versus controls.	(26)
OA	Observational	Plasma	Obese PCOS women have increased OA concentrations compared with women in non-obese PCOS patients and controls.	(48)
OA	Observational	Plasma	OA significantly increased after insulin stimulation in PCOS.	(25)
OA	Observational	FF	The concentration of OA of PCOS with obesity, IR and HA are higher than the control. The concentration of OA of PCOS with IR or HA were higher than PCOS without IR or HA.	(26)
OA	*In vitro*	KGN cell	High-concentration OA stimulation upregulates the transcription levels of IL-6 and IL-8 in through the ERK1/2 signaling pathway, inducing the production of ROS and activation of inflammasomes.	(52)
GA/EA	Observational	Serum	GA increased in non-IR PCOS, and EA increased in non-IR PCOS and decreased in IR PCOS.	(21)

MUFA, monounsaturated fatty acid; FF, follicular fluid; GC–MS, gas chromatography–mass spectrometry; HA-PCOS, hyperandrogenic polycystic ovary syndrome; NA-PCOS, non-hyperandrogenic polycystic ovary syndrome; POA, palmitoleic acid; PA, palmitic acid; OA, oleic acid; SA, stearic acid; SCD, stearoyl-CoA desaturase; MOA, myristoleic acid; IR, insulin resistance; NIR, non-insulin resistance; OGTT, oral glucose tolerance test; HOMA-IR, homeostasis model assessment of insulin resistance; OHSS, ovarian hyperstimulation syndrome; IL-6, interleukin-6; IL-8, interleukin-8; ERK1/2, extracellular signal-regulated kinase 1/2; ROS, reactive oxygen species; GA, gadoleic acid; EA, erucic acid.

#### 3.2.2 Myristoleic acid (MOA, C14:1): a gut microbiota-derived enigma

Myristoleic acid (MOA), a LCFA produced by gut microbiota, demonstrates significant metabolic benefits in preclinical models by improving glycemic control and mitigating adiposity (46, 47). Studies show significantly elevated MOA levels in the follicular fluid (FF) of obese, IR, and HA PCOS groups compared to controls (26), positioning MOA as a key mediator of gut-liver-adipose axis crosstalk. Despite systemic benefits, the pathophysiological relevance of elevated FF MOA in PCOS remains enigmatic. Whether it represents a protective adaptation to ovarian lipotoxicity or a biomarker of dysregulated lipid metabolism warrants investigation through integrated metabolomic and functional studies.

#### 3.2.3 Palmitoleic acid (POA, C16:1) and oleic acid (OA, C18:1): potential compensatory increase

POA and OA, the most abundant monounsaturated fatty acids (MUFAs) in humans, exhibit insulin-axis-associated dysregulation in PCOS (20, 25). These MUFAs are significantly elevated during insulin stimulation in PCOS patients, with overweight individuals showing higher circulating levels than lean counterparts, and IR-PCOS subgroups displaying elevated serum POA concentrations compared to non-IR individuals (18, 24, 48). Notably, POA levels correlate with leptin concentrations, suggesting adipose-hormonal crosstalk in PCOS pathogenesis (20). In the FF, concentrations of both POA and OA are elevated in PCOS patients with concurrent obesity, IR, and HA compared to controls. Specifically, OA concentrations are higher in PCOS patients with IR or HA than in those without these conditions (26). Interestingly, POA has been described as an adipose tissue-derived lipid factor with insulin-sensitizing effects (49). Animal studies demonstrate POA supplementation improves metabolic parameters and exerts anti-inflammatory effects. However, clinical studies report decreased serum POA in PCOS models induced by circadian rhythm disruption (50). Similarly, OA exhibits anti-inflammatory and antioxidant properties counteracting SFAs lipotoxicity and supporting oocyte development through: metabolic partitioning of fatty acids, membrane structural modifications, OS attenuation, and intracellular signaling regulation (51). Conversely, *in vitro* studies show high-concentration OA upregulates IL-6 and IL-8 transcription in KGN cells via ERK1/2 signaling, leading to ROS production and inflammasome activation (52). These seemingly contradictory findings-OA exhibiting both cytoprotective and pro-inflammatory effects-likely reflect a dose-dependent biphasic action, a common phenomenon for bioactive lipids. At physiological or moderately elevated concentrations, OA may function as a compensatory mediator to counteract SFA-induced lipotoxicity and maintain cellular homeostasis. However, when OA accumulates excessively-as observed in the FF of obese, insulin-resistant, or HA-PCOS patients. It may surpass a pathological threshold, triggering ERK1/2-mediated inflammatory responses and oxidative stress. This concentration-dependent duality suggests that the net effect of OA in PCOS depends on the local lipid milieu and the degree of metabolic dysregulation. Future studies should define the critical concentration range at which OA transitions from protective to detrimental within the ovarian microenvironment.

#### 3.2.4 Compensatory adaptation and therapeutic potential of MUFAs in PCOS

Studies report elevated total MUFA levels in the serum of PCOS patients (18), though levels are lower in reproductive subtypes than metabolic subtypes (20). Serum MUFAs concentrations progressively decrease from controls to non-IR PCOS and further to IR PCOS (21). Furthermore, FF MUFAs levels are markedly increased in HA-PCOS women compared to controls, an alteration absent in non-hyperandrogenic (NA)-PCOS groups (22). Elevated MUFAs in PCOS may partially stem from dietary factors; girls with PCOS consume more MUFAs, and MUFA intake positively correlates with PCOS risk (53). Paradoxically, clinical trials indicate that MUFA-rich diet increases adiponectin and reduces free androgen index (54). MUFAs may exert cytoprotective effects by counteracting SFA-induced endoplasmic reticulum (ER) stress (55). Human and animal studies demonstrate that substituting SFAs with MUFAs activates anti-inflammatory mechanisms and reverses SFA-induced adipose tissue dysfunction (45). Clinical and preclinical evidence on MUFAs in PCOS yields conflicting results: while MUFA-rich supplementation improves metabolic phenotypes (56), specific MUFAs are elevated in blood and ovarian tissues of PCOS patients. These paradoxes suggest that increased circulating MUFAs may represent a compensatory adaptation to excessive SFA intake and resultant lipotoxicity. Exogenous MUFA consumption could ameliorate PCOS by balancing unsaturated/saturated fatty acid ratios or inhibiting stearoyl-CoA desaturase 1 (SCD1) activity. Nevertheless, whether specific MUFAs confer phenotype-specific therapeutic benefits requires further investigation.

### 3.3 The role of PUFAs in PCOS: altered profiles, inflammatory-metabolic crosstalk, and therapeutic opportunities

#### 3.3.1 PUFAs profiles as metabolic biomarkers in PCOS heterogeneity

**Figure 4:**
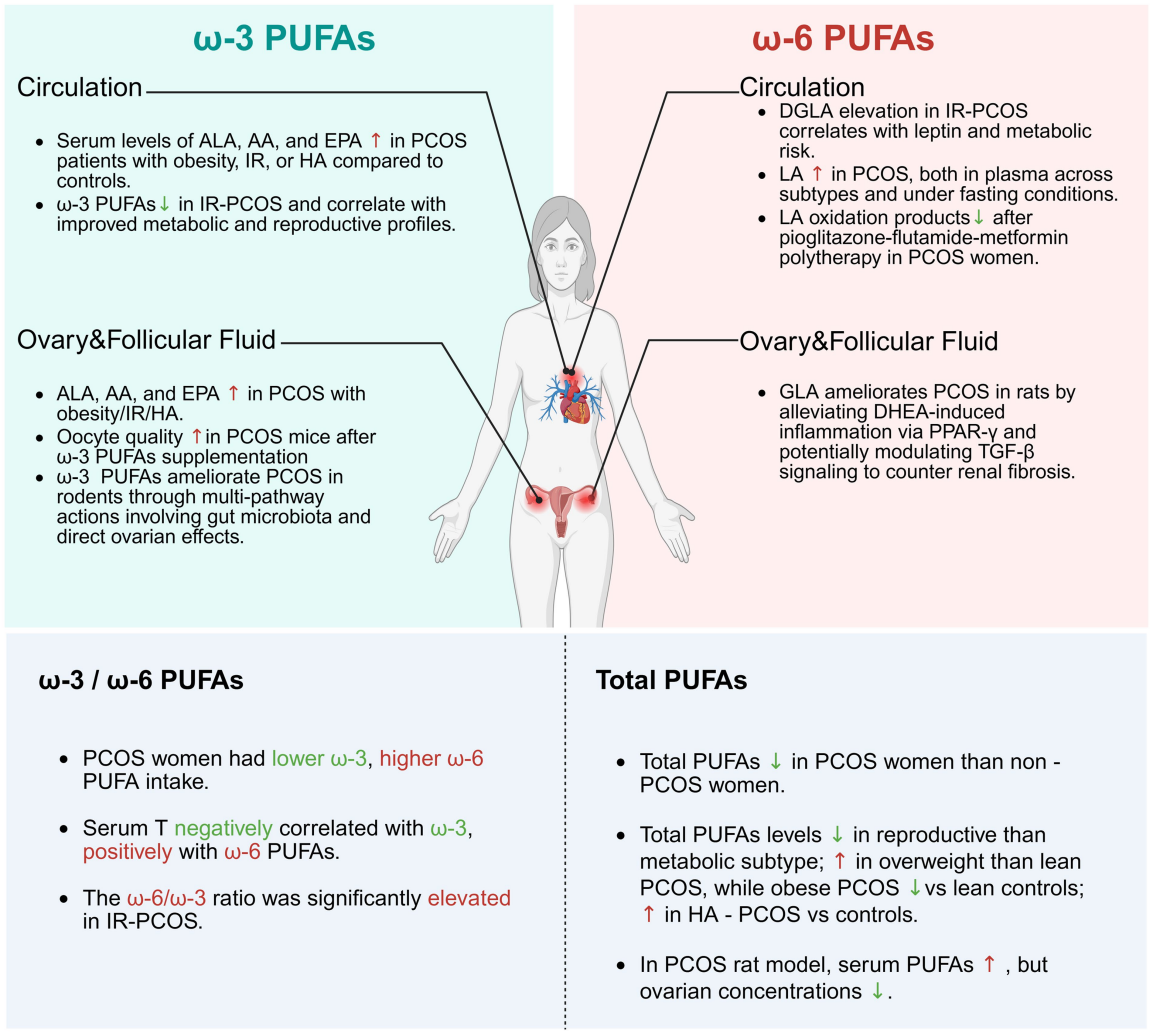
Polyunsaturated fatty acids in PCOS: metabolic imbalance, reproductive dysfunction, and therapeutic implications (Created with BioRender.com, agreement number: XZ29VKF5SV). This integrated schematic illustrates the dysregulation of omega-3 (*ω*-3) and omega-6 (ω-6) polyunsaturated fatty acids (PUFAs) in polycystic ovary syndrome (PCOS), highlighting alterations across circulation, ovarian tissue, and follicular fluid, as well as their implications for metabolic and reproductive health. In circulation, serum levels of α-linolenic acid (ALA), arachidonic acid (AA), and eicosapentaenoic acid (EPA) are elevated in PCOS patients with obesity, insulin resistance (IR), or hyperandrogenism (HA) compared to controls, whereas total ω-3 PUFAs are reduced in IR-PCOS and correlate with improved metabolic and reproductive outcomes. In contrast, dihomo-γ-linolenic acid (DGLA) is elevated in IR-PCOS and associated with increased leptin and metabolic risk; linoleic acid (LA) is elevated in all PCOS subtypes under both fasting and fed conditions, and its oxidation products decrease after pioglitazone–flutamide–metformin polytherapy. In ovary and follicular fluid, ALA, AA, and EPA are increased in PCOS with obesity/IR/HA; oocyte quality improves in PCOS mice following ω-3 PUFA supplementation, and ω-3 PUFAs ameliorate PCOS in rodents via multi-pathway mechanisms involving gut microbiota modulation and direct ovarian effects. Gamma-linolenic acid (GLA) alleviates DHEA-induced inflammation in rats through PPAR-γ activation and may counteract renal fibrosis via TGF-*β* signaling. The ω-3/ω-6 PUFA balance is significantly disrupted in PCOS: women exhibit lower ω-3 and higher ω-6 PUFA intake, with serum testosterone negatively correlated with ω-3 and positively with ω-6 PUFAs; the ω-6/ω-3 ratio is markedly elevated in IR-PCOS. Total PUFAs are reduced in PCOS women compared to non-PCOS controls, with further decreases in reproductive versus metabolic subtypes and in overweight versus lean PCOS, while obese PCOS shows reduced total PUFAs versus lean controls but increased levels in HA-PCOS. In a PCOS rat model, serum PUFAs increase, yet ovarian concentrations decrease, indicating compartmentalized lipid dysregulation. Collectively, these findings underscore a pro-inflammatory, pro-lipotoxic lipid profile in PCOS characterized by an imbalance between ω-3 and ω-6 PUFAs, with potential therapeutic relevance for dietary and pharmacological interventions targeting lipid metabolism.

PUFAs are fatty acids containing two or more carbon–carbon double bonds in their acyl chain. This review focuses on long-chain PUFAs (typically with chain lengths of C18 to C22), which are the most abundant and biologically active forms in mammalian systems and are critically implicated in PCOS pathophysiology. They are categorized into omega-3 (e.g., *α*-linolenic acid, ALA) and omega-6 (e.g., linoleic acid, LA) series based on the position of the first double bond from the methyl terminus. As essential nutrients, ALA and LA must be obtained dietarily and are metabolized via FADS gene-encoded Δ5/Δ6 desaturases to yield bioactive derivatives: eicosapentaenoic acid (EPA), docosahexaenoic acid (DHA), and docosapentaenoic acid (DPA) from ALA; *γ*-linolenic acid (GLA), dihomo-γ-linolenic acid (DGLA), and arachidonic acid (AA) from LA. FADS1/2 polymorphisms (e.g., rs174570) correlate with altered PUFA profiles and elevated polycystic ovary syndrome (PCOS) risk, indicating genetic regulation of PUFA metabolism (57). These metabolites exhibit distinct, often antagonistic functions: n-3 PUFAs demonstrate anti-inflammatory, insulin-sensitizing, and cardiometabolic protective effects, whereas n-6 PUFAs (particularly AA derivatives) promote pro-inflammatory response (58). Abnormal PUFAs profiles have been consistently observed in women with PCOS across different tissues, including serum, FF, and adipose tissue (59–61). In high-fat diet and androgen-induced PCOS rat models, serum levels of PUFAs are increased, while their concentrations in ovarian tissue reduce (62). Clinical studies report altered serum PUFA levels characterized by lower n-3 and elevated n-6 levels, resulting in an increased n-6/n-3 ratio compared with healthy controls (63). In FF, elevated levels of specific PUFAs (e.g., AA and EPA) and their metabolites have been documented, particularly in hyperandrogenic and insulin-resistant PCOS subtypes, potentially impairing oocyte quality (59, 64). Differential metabolic patterns are evident across PCOS phenotypes. Insulin-resistant and obese individuals exhibit more pronounced PUFA metabolism disturbances, underscoring the link between metabolic status and dysregulated lipid profiles (61, 65). Current advances in research on PUFAs and PCOS are summarized in Figure 4 and Table 3.

**Table 3:** Summary of the association between PUFA and PCOS.

LCFA	Research Type	Sample	Conclusion	Ref
n-3/n-6 PUFA	Observational	Serum	The main source of PUFAs is dietary intake, women with PCOS had lower intakes of n-3 PUFAs and higher intakes of n-6 PUFAs, and serum testosterone levels were significantly negatively correlated with n-3 PUFA and positively correlated with n-6 PUFA.	(63)
n-3/n-6 PUFA	Observational	Serum	n-6 PUFA/n-3 PUFA ratio increased significantly in IR PCOS patients compared with non-IR PCOS and controls.	(21)
n-3/n-6 PUFA	*In vivo*	Rat	The appropriate n-3/n-6 PUFAs ratio significantly increased the ovarian weight and improved the ovarian structure of DHT-induced PCOS rats by increasing the expression of steroidogenesis enzymes, which are related to hormone secretion and ovarian functions.	(82)
Total PUFA	Observational	Serum	The levels of PUFAs in the reproductive subtype of PCOS were lower than those in the metabolic subtype.	(20)
Total PUFA	Observational	Serum	PUFAs intake young women with PCOS is significantly positively correlated with free testosterone.	(65)
Total PUFA	Observational	Serum	Obese patients with PCOS had decreased PUFA levels vs. lean controls.	(19)
Total PUFA	Observational	Serum	Compared with lean PCOS, overweight PCOS have higher level of PUFAs.	(18)
Total PUFA	Observational	FF	Compared with the controls, the levels of PUFAs of HA-PCOS women were dramatically increased, while this alteration was not visible in the NA-PCOS group.	(22)
Total PUFA	Observational	Subcutaneous adipose tissue	The total PUFAs and the levels of ALA and EPA of PCOS women was lower than that of non-PCOS women.	(61)
Total PUFA	*In vivo*	Rat	In high-fat diet and androgen-induced PCOS rat models, serum levels of PUFA are increased, while their concentrations in ovarian tissue reduce.	(62)
n-3 PUFA	Observational	Serum	Total n-3 PUFA levels, DPA, DHA were significantly lower in IR PCOS patients compared with non-IR PCOS and controls.	(21)
n-3 PUFA	Observational	Serum	The levels of ALA, EPA increase in PCOS, but there was no significant difference between IR-PCOS and NIR-PCOS.	(24)
n-3 PUFA	Observational	Serum	Both dietary and serum n-3 PUFA, mainly EPA and DPA, were negatively correlated with PCOS-related parameters, such as BMI, fasting insulin, total testosterone and hsCRP, but positively correlated with FSH and SHBG.	(69)
n-3 PUFA	Observational	FF	ALA, AA and EPA levels of PCOS with obesity, IR or HA were all higher than those in the control.	(26)
n-3 PUFA	*In vivo*	Mouse	N-3 PUFAs supplementation ameliorate PCOS mice by modulating gut microbiota and alleviating ovarian dysfunction and insulin resistance. Moreover, n-3 PUFAs may have direct effects on the ovary to inhibit inflammation.	(85)
n-3 PUFA	*In vivo*	Mouse	Adding n-3 PUFAs to the diet has little effect on the body weight of PCOS mice, but it greatly alleviates the disorder of blood hormone levels in PCOS mice, improves the estrous cycle and ovarian function of PCOS mice, and promotes ferroptosis of ovarian granulosa cells.	(77)
n-3 PUFA	*In vivo*	Rat	Both natural fish oil rich in n-3 PUFAs and synthetic n3-PUFA mixtures can improve blood lipids and hyperglycemia, reduce body weight and histopathological damage in PCOS rats.	(87)
n-3 PUFA	*In vitro*	KGN cell	The n-3 PUFA treatment can inhibit the YAP1 (Yes-associated protein 1)/Nrf2(NF-E2-related factor 2) crosstalk that suppresses over proliferating ovarian granulosa cells and promotes ferroptosis.	(77)
n-3 PUFA	*In vitro*	Mouse primary oocyte	The oocyte maturation rate of PCOS mice significantly increased and the abnormal rates of spindles and chromosomes were lower after cultured *in vitro* with n-3 PUFAs, and maybe describe to the rescued expression of an antioxidant-related gene and DNA damage repair genes.	(83)
AA	Observational	FF	AA levels of PCOS patients are higher, and the levels of and AA metabolites were also significantly higher.	(60)
AA	*In vivo*	Rat	AA levels of the PCOS group in ovarian tissues were significantly decreased while the levels in serum increased compared to those in the controls.	(62)
AA	*in vitro*	KGN cell	AA may induce oxidative stress (OS) and upregulate the expression of GDF15 as a response to OS.	(60)
ALA	*In vivo*	Rat	ALA-rich oil supplement improved the reproductive and metabolic phenotypes of PCOS rats, and improved the inflammatory status of plasma and ovaries, significantly modulated the composition of intestinal flora and vaginal microbiota. Plasma lipopolysaccharide (LPS) levels decrease and SCFAs increase.	(84)
GLA	*In vivo*	Rat	Oral administration of n-6 PUFA: GLA reverses the increase in the expression of TNFα, IL-1α, and MCP-1 in the periovarian adipose tissue of DHEA-induced PCOS rats.	(74)
GLA	*In vivo*	Rat	GLA is able to reduce the inflammatory response due to DHEA stimulation and thereafter potentially attenuate PCOS via the PPAR-γ pathway.	(75)
GLA	*In vivo*	Rat	The DHEA-induced PCOS model shows a possible development of renal fibrosis, the GLA might act as a ligand to regulate TGF-β signaling in glomerulosclerosis in a DHEA-induced PCOS model.	(76)
DGLA	Observational	Serum	DGLA is associated with insulin-related metabolic risk factors and is significantly positively correlated with leptin levels in women with PCOS.	(20)
DGLA	Observational	Serum	DGLA increased significantly in IR PCOS patients compared with non-IR PCOS and controls.	(21)
LA	Observational	Plasma	Plasma LA levels in PCOS subtypes A, B, C, and D were all higher than those in control women.	(9)
LA	Observational	Plasma	Fasting LA level significantly increase in PCOS.	(25)
LA	RCT	Serum	Levels of LA oxidation products, were significantly reduced in women with PCOS after pioglitazone-flutamide-metformin polytherapy.	(118)

PUFA, polyunsaturated fatty acid; PCOS, polycystic ovary syndrome; IR, insulin resistance; DHT, dihydrotestosterone; FF, follicular fluid; HA, hyperandrogenism; NA, non-hyperandrogenism; ALA, alpha-linolenic acid; EPA, eicosapentaenoic acid; DHA, docosahexaenoic acid; DPA, docosapentaenoic acid; AA, arachidonic acid; GLA, gamma-linolenic acid; DGLA, dihomo-γ-linolenic acid; LA, linoleic acid; hsCRP, high-sensitivity c-reactive protein; SHBG, sex hormone-binding globulin; FSH, follicle-stimulating hormone; YAP1, yes-associated protein 1; Nrf2, NF-E2-related factor 2; GDF15, growth differentiation factor 15; TNFα, tumor necrosis factor alpha; IL-1α, interleukin-1 alpha; MCP-1, monocyte chemoattractant protein-1; PPAR-γ, peroxisome proliferator-activated receptor gamma; TGF-β, transforming growth factor beta; LPS, lipopolysaccharide; SCFAs, short-chain fatty acids; NMR, nuclear magnetic resonance; RCT, randomized controlled trial; DHEA, dehydroepiandrosterone; OS, oxidative stress; KGN, human ovarian granulosa cell line.

#### 3.3.2 PUFA subtypes and their functional implications in PCOS

*ω*-3 PUFAs play beneficial roles in alleviating insulin resistance, reducing inflammation, and improving metabolic and reproductive parameters in PCOS (66–69). EPA and DHA supplementation has shown effectiveness in clinical studies, improving insulin sensitivity, decreasing inflammatory markers, and enhancing ovulatory function (70–72). Specifically, DHA contributes to follicular maturation by regulating apoptosis, autophagy, and lipid droplet metabolism within ovarian GCs, thus preserving ovarian health and function.

In contrast to *ω*-3 PUFAs, ω-6 PUFAs, particularly AA and its metabolites, promote pro-inflammatory pathways. Elevated AA-derived eicosanoids may exacerbate chronic low-grade inflammation characteristic of PCOS, disrupting ovarian follicle maturation and reducing oocyte quality (73). Excess AA within the follicular microenvironment has been associated with detrimental effects on ovarian function, potentially impairing fertility outcomes (59, 60). However, research has demonstrated that not all n-6 PUFAs exert detrimental effects on PCOS. Studies indicate that GLA exhibits anti-inflammatory and anti-fibrotic effects in PCOS animal models (74–76). This suggests that the effects of n-6 PUFAs on PCOS may be contingent upon their specific subtypes and distinct pathophysiological contexts.

#### 3.3.3 Dual roles of PUFAs in PCOS pathogenesis: n-3/n-6 imbalance modulates inflammation, ferroptosis, lipid metabolism, and ovarian signaling pathways

PUFAs play an intricate role in modulating inflammatory and metabolic pathways implicated in PCOS pathology. ω-3 PUFAs generally exert anti-inflammatory properties by inhibiting pro-inflammatory cytokines and modulating NF-κB signaling, whereas ω-6 PUFAs-particularly AA-are typically pro-inflammatory. This highlights the significance of maintaining an optimal ω-6/ω-3 ratio to manage inflammation in PCOS (58, 63). Additionally, DHA specifically modulates ferroptosis, an iron-dependent form of oxidative cell death that is increasingly recognized in ovarian dysfunction associated with PCOS (77, 78). Dysregulated PUFA metabolism also contributes to disturbances in lipid metabolism by influencing lipid droplet formation, regulating the expression of FASN, and activating sterol regulatory element-binding proteins (SREBPs), further exacerbating metabolic anomalies in PCOS (79). Moreover, PUFAs regulate pivotal signaling pathways such as AKT/AMPK and the YAP1-Nrf2 axis, significantly influencing ovarian cell proliferation, differentiation, and stress responses (77).

#### 3.3.4 Therapeutic insights from PUFA supplementation

Emerging clinical and preclinical evidence supports the therapeutic potential of PUFA supplementation in PCOS management. Clinical trials have shown that EPA and DHA supplementation effectively enhances insulin sensitivity, optimizes lipid profiles, reduces inflammatory markers, and improves ovulatory function in women with PCOS (71, 80, 81). Animal model studies further elucidated the multifaceted benefits of PUFA intervention, highlighting improvements in metabolic disturbances, inflammatory states, and ovarian dysfunction (82, 83). Specifically, n-3 PUFA supplementation has been found to alleviate hyperandrogenism, improve ovarian morphology, normalize adipokine profiles such as leptin and adiponectin, and beneficially modulate gut microbiota composition (84–87). Consequently, optimizing PUFA intake, particularly through increased consumption of n-3 PUFAs combined with balanced n-6 intake, emerges as a promising nutritional strategy to ameliorate PCOS-related metabolic, inflammatory, and reproductive dysfunctions. However, further research is essential to refine these dietary strategies and establish personalized recommendations for effective PCOS management (63).

## 4 LCFAs membrane receptor and PCOS: transporting and signaling

LCFAs require specific receptors to exert physiological functions. These receptors can be categorized by subcellular localization into nuclear receptors and membrane receptors. LCFAs regulate metabolic and gene regulatory networks primarily through nuclear receptors, particularly PPARs (17). Meanwhile, membrane receptors mediate LCFA transport and signal transduction (10). This section focuses on recent advances in understanding LCFA membrane receptors—including transport receptors (fatty acid-binding proteins [FABPs], fatty acid transport proteins [FATPs], and CD36) and G protein-coupled receptors (GPCRs; GPR40/120) and their implications in PCOS (88).

### 4.1 LCFA transport receptors: FABPs, CD36, FATPs

LCFAs are hydrophobic molecules, and the transfer of LCFAs through the plasma membrane remains highly controversial. Current evidence indicates that LCFAs require specialized transport mechanisms to cross biological membranes. LCFAs enter cells via passive diffusion and protein-mediated translocation, involving key transporters like FABPs, FATPs, and CD36. In PCOS, disrupted lipid metabolism, characterized by altered expression of LCFA transporters and receptors, contributes to IR, HA, and ovarian dysfunction. Understanding the role of LCFA transport receptors in these pathways may provide novel therapeutic targets for managing the metabolic and endocrine features of PCOS.

#### 4.1.1 FABPs and PCOS: genetic associations, tissue-specific roles, and metabolic implications

FABPs are lipid-binding proteins that play a central role in fatty acid metabolism. At least nine mammalian FABPs (FABP1-9) have been identified, each exhibiting tissue-specific expression patterns and highly conserved tertiary structures (89). FABPs function primarily as cytoplasmic lipid chaperones, binding to both saturated and unsaturated fatty acids (SFAs and UFAs), facilitating their solubilization, transport, and metabolism. In addition to intracellular roles, FABPs may be secreted extracellularly, influencing local and systemic metabolic processes and inflammation. FABP gene polymorphisms are closely associated with PCOS. Genome-wide association studies (GWAS) have identified significant associations between single nucleotide polymorphisms (SNPs), such as rs2197076 and rs2241883 located in the FABP1 gene, and PCOS. Notably, rs2197076 has been shown to play a particularly prominent role in the pathogenesis of this condition. These genetic variations highlight the involvement of FABPs in PCOS development, reinforcing their biomarker potential (90). Current research predominantly focuses on FABP4 and FABP5 in PCOS; the following sections review emerging evidence on their pathogenic links to this disorder.

FABP4 (A-FABP): FABP4, also known as adipose FABP (A-FABP), is involved in both intracellular fatty acid transport and systemic metabolic regulation when secreted into the bloodstream. Research has demonstrated that serum FABP4 levels in women with PCOS correlate positively with BMI, HOMA-IR, and testosterone levels, while inversely correlating with HDL-C (91, 92). Notably, FABP4 levels may predict long-term treatment response in PCOS (93). However, FABP4’s direct impact on insulin resistance, inflammation, and HA remains inconclusive. Some studies implicate its involvement, but effects lack consistent significance. Intriguingly, after BMI adjustment, serum FABP4 levels and FABP4 mRNA expression in GCs of PCOS patients remain elevated versus controls (94), suggesting FABP4 may exert obesity-independent pathological effects in PCOS pathogenesis.

Furthermore, FABP4 overexpression occurs in abdominal subcutaneous adipose stem cells from PCOS women, with significant increases in total fatty acid content and *de novo* fatty acid synthesis. Adipogenesis-related genes (including FABP4) are up regulated during differentiation (95). These findings imply PCOS might accompanies FABP4 overexpression and secretion, independent of obesity. Critically, insulin-sensitizing and lipid-lowering therapies fail to significantly reduce FABP4 levels in PCOS (96), indicating FABP4 may promote disease progression through alternative pathways.

FABP5 (E-FABP): FABP5, also termed epidermal fatty acid-binding protein (E-FABP), is upregulated in ovarian GCs of PCOS patients and animal models (97). *In vitro* studies demonstrate that FABP5 promotes fatty acid synthesis and excessive GC proliferation by activating the PI3K-AKT signaling pathway (98). While these *in vitro* findings suggest a potential role for the FABP5-PI3K-AKT axis in GC dysfunction, it remains to be determined whether this mechanism represents a causal driver of PCOS pathogenesis or a secondary adaptive response to the underlying metabolic and hormonal disturbances. Current evidence is primarily derived from correlation analyses in clinical samples and loss-of-function studies in cultured cells which cannot establish causality in the context of the complex *in vivo* PCOS milieu. Whether FABP5 overexpression in GC actively contributes to follicular arrest and anovulation, or merely reflects a compensatory response to local lipotoxicity or hyperandrogenemia, requires validation through FABP5 knockout or overexpression studies in PCOS animal models. Such in vivo approaches would help distinguish the FABP5-PI3K-AKT axis as a potential therapeutic target versus an epiphenomenon of disease progression.

Collectively, accumulating evidence associates FABP over expression—particularly of FABP4 and FABP5—with metabolic and inflammatory dysregulation in PCOS. These findings underscore FABPs’ dual utility as PCOS biomarkers and therapeutic targets. Further research should explore FABP-targeted interventions to fully delineate their clinical potential.

#### 4.1.2 CD36 and PCOS: hormonal regulation, tissue-specific expression, and pathophysiological implications

CD36 is a member of the class B scavenger receptor family and plays a significant role in LCFAs and lipid transport. Beyond LCFAs, CD36 binds multiple ligands including oxidized low-density lipoprotein (oxLDL), thrombospondin, advanced glycation end products (AGEs), and other lipid-protein complexes (99). CD36 is widely expressed in numerous tissues, participating in pathophysiological processes such as immune and metabolic regulation. The crystal structure of CD36 reveals a molecular LCFA transport channel, with its synthesis, distribution, and function regulated by post-transcriptional modifications including ubiquitination, glycosylation, phosphorylation, and palmitoylation. Palmitoylated CD36 functions as a lipid saturation sensor, promoting selective uptake of MUFAs to prevent SFAs-induced lipotoxicity (100). Additionally, CD36 interacts with membrane receptors, responds to extracellular signals, and forms signal transduction complexes that activate downstream effectors.

In PCOS, obesity disrupts adipose metabolism and impairs granulosa cell (GC) function. Elevated CD36 protein levels in adipose tissue positively correlate with fasting serum insulin, insulin resistance, and testosterone dysregulation (101). CD36 expression in GCs is significantly higher in PCOS patients compared to controls and is upregulated in obese women with or without PCOS relative to lean non-PCOS individuals (102, 103). *In vitro*, high testosterone and insulin induce CD36 mRNA expression, while the PPARγ agonist rosiglitazone further enhances this effect in a dose-dependent manner, indicating hormonal modulation (102).

However, inconsistent findings exist: CD36 mRNA is decreased in PCOS theca cells and negatively correlates with testosterone and insulin in FF (104). Dihydrotestosterone (DHT)-treated PCOS rats show reduced CD36 in cardiac plasma membranes and intracellular pools (105), decreasing fatty acid uptake and potentially increasing cardiovascular risk. CD36 is not only present on the cell membrane but also circulates in the plasma as soluble CD36 (sCD36). sCD36 is negatively associated with insulin sensitivity in PCOS; pioglitazone lowers sCD36 while improving insulin-stimulated glucose metabolism (106). The mechanisms underlying altered CD36 expression and its pathophysiological impacts require further investigation.

In summary, CD36 critically regulates fatty acid metabolism, immunity, and metabolic homeostasis. Its overexpression in PCOS adipose tissue and GCs, associated with hormonal dysregulation, suggests contributions to metabolic-endocrine disturbances. Mechanistic studies and therapeutic potential of CD36 targeting warrant further exploration.

#### 4.1.3 FATPs isoforms in metabolic diseases: dual transport-enzyme functions and PCOS knowledge gaps

The FATPs family, also known as the solute carrier family 27 (SLC27), consists of six members (FATP1-6), encoded by SLC27A1–6. These proteins share sequence similarities, and although they can functionally substitute for each other, each FATP member exhibits a distinct expression profile and role in specific tissues. FATPs are proposed to act as direct LCFA transporters and also catalyze their activation by converting them into acyl-CoA thioesters via intrinsic acyl-CoA synthetase (ACS) activity. The resulting acyl-CoA derivatives are then utilized in various metabolic processes or functionally serve as bifunctional proteins with both transport and enzymatic activity (107). The FATP family serves as a central molecular hub in metabolic diseases such as obesity and NAFLD, regulating cellular fatty acid uptake and metabolic flux (108, 109). However, their investigation in ovarian biology and PCOS remains nascent. Current evidence is sparse: for instance, FATP4, critical for intestinal lipid absorption, may have implications for the nutrient supply to the ovary, and preliminary data suggest detectable expression of certain FATP isoforms in ovarian tissue. However, a systematic analysis of their expression patterns across ovarian cell types (granulosa, theca, oocyte) in healthy versus PCOS states, and their potential synergistic or regulatory relationships with other key transporters like CD36 and FABP4/5 in the ovary, are entirely unexplored. This constitutes a significant gap in understanding the complete picture of fatty acid handling in PCOS pathophysiology. Elucidating FATP functions in the ovary represents a critical future direction, as they could serve as novel nodal points integrating dietary lipid availability with follicular development and steroidogenesis.

### 4.2 FFAR1/FFAR4 (GPR40/120) signaling in PCOS: ligand selectivity, metabolic dysregulation, and therapeutic targeting

In addition to FAs transport receptors, LCFAs also activate a group of GPCRs on the cell membrane, known as free fatty acid receptors (FFARs). Within the FFARs family, FFAR1 (GPR40) and FFAR4 (GPR120) are classified as LCFAs receptors and play critical roles in metabolism and energy regulation. FFAR1 and FFAR4 are expressed in various tissues, including the pituitary gland, intestine, adipose tissue, and macrophages. These receptors exert multiple metabolic effects, such as promoting insulin sensitization, stimulating glucagon-like peptide-1 (GLP-1) secretion, and regulating lipogenesis. Within the FFARs family, FFAR1 and FFAR4 are activated by medium- and long-chain fatty acids. FFAR1 can be co-activated by both saturated and unsaturated fatty acids: unsaturated fatty acids (e.g., DHA, EPA, OA) and saturated fatty acids of specific chain lengths activate FFAR1 at micromolar concentrations. The activation of FFAR1 by saturated fatty acids demonstrates a significant chain length dependence, with PA (C16:0) being the most potent saturated ligand (88, 110–112). In contrast, FFAR4 exhibits higher affinity for long-chain polyunsaturated fatty acids (particularly n-3 PUFA), but can also be activated by saturated fatty acids at higher concentrations (112). This selectivity stems from the unique spatial configuration of double bonds in UFAs, which engage specific amino acid residues in FFAR4 to initiate downstream signaling.

In the context of PCOS, the role of FFAR4 (GPR120) has garnered attention. FFAR4 protein levels are downregulated in the liver and ovaries of PCOS rat models. Treatment with FFAR4 agonists improves hepatic lipid metabolism, reduces body weight, enhances ovarian function, and ameliorates IR, suggesting that targeting FFAR4 may be an effective strategy for managing PCOS (113). Some studies also suggest that ovarian hormones may modulate FFAR4 expression in the mouse pituitary gland (114). Additionally, FFAR4 influences pregnancy outcomes in PCOS patients: its expression is downregulated in the decidua of women with spontaneous abortion, and animal studies demonstrate that FFAR4 activation ameliorates LPS-induced abortion by enhancing ERK/AMPK/FOXO1 signaling and promoting GLUT1-mediated glucose uptake to support decidualization (115). Similar to FFAR4 (GPR120), FFAR1 (GPR40) also plays a significant role in energy metabolism and inflammatory immunity (116, 117).

Although both are LCFA-sensing GPCRs, FFAR1 (GPR40) and FFAR4 (GPR120) differ in ligand preference, signaling bias, and tissue distribution, suggesting potential for complex complementary, synergistic, or even antagonistic relationships. For example, in pancreatic *β*-cells, FFAR1 activation primarily amplifies glucose-stimulated insulin secretion, whereas FFAR4 potentiates GLP-1 release; their co-activation could theoretically fine-tune insulin response. In macrophages, both receptors can mediate anti-inflammatory effects, but through distinct pathways (e.g., FFAR4 via β-arrestin2/NF-κB, FFAR1 via alternative mechanisms), suggesting possible synergy in resolving inflammation. Conversely, in tissues where they are co-expressed (e.g., adipocytes, enteroendocrine cells), competition for common ligands or differential coupling to downstream effectors could result in context-dependent antagonism or biased signaling. In PCOS, where chronic inflammation and insulin resistance are hallmarks, understanding whether the dysregulated LCFA pool disproportionately activates one receptor over the other, or if a loss of balance between FFAR1 and FFAR4 signaling contributes to pathology, is a compelling unanswered question. Exploring this receptor crosstalk could reveal novel mechanisms and inform the development of dual-targeting or balanced agonist therapies.

However, limited research has addressed its specific role in PCOS. Given that FFAR1/GPR40 and FFAR4/GPR120 are key mediators of fatty acid metabolism and energy regulation—both central to PCOS pathophysiology—targeting these receptors may represent a novel therapeutic approach. Nonetheless, further research is necessary to better understand the intricate mechanisms through which these receptors influence PCOS and to explore their full therapeutic potential.

## 5 Conclusions and future perspectives

**Figure 5:**
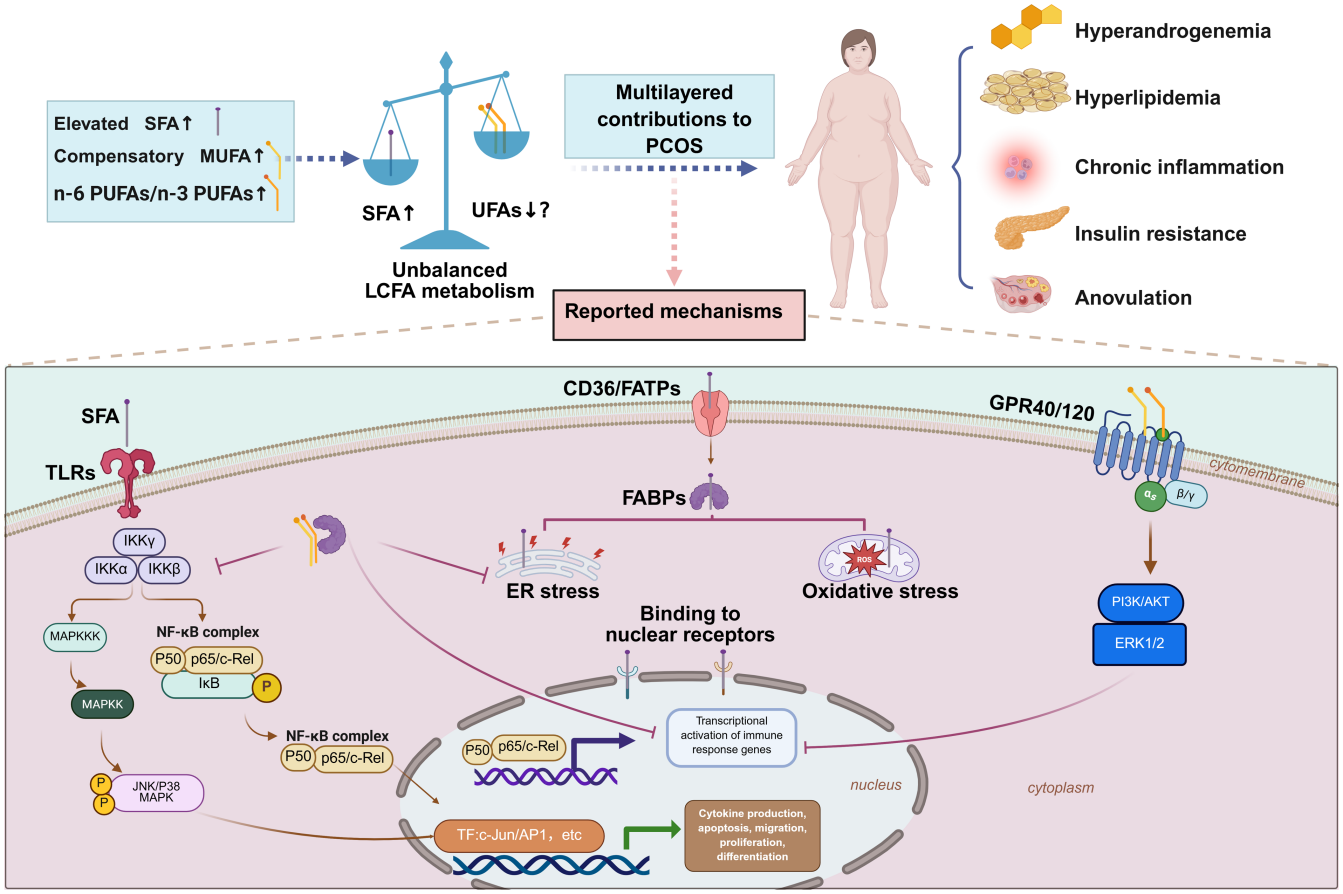
Pathogenesis of long-chain fatty acids (LCFAs) and receptors for LCFAs in polycystic ovary syndrome (PCOS) (Created with BioRender.com, agreement number: PI29VKF7BM). This integrated schematic illustrates the central role of unbalanced long-chain fatty acid (LCFA) metabolism in the pathogenesis of polycystic ovary syndrome (PCOS), highlighting how alterations in saturated fatty acids (SFAs), monounsaturated fatty acids (MUFAs), and polyunsaturated fatty acids (PUFAs)—particularly the n-6/n-3 ratio—contribute to key clinical features including hyperandrogenemia, hyperlipidemia, chronic inflammation, insulin resistance, and anovulation. At the systemic level, elevated SFAs are associated with compensatory increases in MUFAs and a disrupted n-6/n-3 PUFA balance, leading to impaired LCFA homeostasis. This metabolic dysregulation is associated with multilayered contributions to PCOS through multiple cellular signaling pathways. Intracellularly, SFAs activate Toll-like receptors (TLRs), triggering the IKK complex and downstream NF-κB activation via MAPK cascades, promoting pro-inflammatory cytokine production and immune gene transcription. SFA uptake via CD36/FATPs leads to endoplasmic reticulum (ER) stress and oxidative stress, mediated by mitochondrial ROS generation. Fatty acid-binding proteins (FABPs) facilitate intracellular trafficking and modulate nuclear receptor signaling, influencing gene expression related to proliferation, differentiation, and apoptosis. Additionally, SFAs bind GPR40/120 on the plasma membrane, activating PI3K/AKT and ERK1/2 pathways, which may contribute to insulin resistance and ovarian dysfunction. These interconnected mechanisms underscore how lipid imbalance initiates and sustains inflammatory, metabolic, and reproductive derangements in PCOS, positioning LCFA metabolism as a critical node for therapeutic intervention. LCFA, long-chain fatty acid; SFA, saturated fatty acid; MUFA, monounsaturated fatty acid; PUFA, polyunsaturated fatty acid.

In conclusion, PCOS is a prevalent endocrine disorder characterized by the coexistence of reproductive and metabolic disturbances. Its clinical manifestations are heterogeneous, and its pathogenesis remains incompletely understood. Imbalances among SFAs, MUFAs, and PUFAs strongly correlate with PCOS phenotypes, including chronic inflammation, IR, ER stress, and dyslipidemia. The intricate interplay between LCFAs and their membrane receptors constitutes a pivotal axis in PCOS pathogenesis, bridging metabolic dysregulation, chronic inflammation, and reproductive dysfunction. The pathogenic mechanisms involving LCFAs and LCFAs receptors in PCOS are summarized in Figure 5. SFAs accumulate in serum/follicular fluid of IR/HA-PCOS phenotypes, inducing lipotoxicity via TLR/NF-κB inflammation, mitochondrial ROS, and ER stress. Paradoxically, their role in protein palmitoylation may modulate immune-metabolic crosstalk. MUFAs (e.g., POA, OA) exhibit compensatory elevation but context-dependent roles: while OA counteracts SFA-induced ER stress, high concentrations activate pro-inflammatory ERK signaling in granulosa cells. PUFAs display divergent effects: n-3 PUFAs (EPA/DHA) improve insulin sensitivity and ovarian function, whereas n-6 PUFAs (e.g., AA) promote inflammation and ferroptosis. The elevated n-6/n-3 ratio emerges as a key biomarker of PCOS severity. Transport receptors (FABP4/5, CD36, FATPs) are dysregulated in adipose/ovarian tissues, driving IR and hyperandrogenism independently of obesity. FABP4 overexpression correlates with HOMA-IR and testosterone levels, while CD36 palmitoylation acts as a lipid saturation sensor. Signaling receptors (FFAR4/GPR120, FFAR1/GPR40) demonstrate phenotype-specific alterations: FFAR4 downregulation in PCOS models exacerbates metabolic dysfunction, whereas its agonists improve hepatic lipid metabolism and ovarian function (113).

To translate mechanistic insights into clinical practice, future research must address these critical frontiers:

1. Resolving Spatial and Temporal Heterogeneity: future research must prioritize resolving spatial and temporal heterogeneity in ovarian lipid metabolism. This requires mapping the distribution of LCFAs across ovarian subcompartments-such as theca versus GCs-using single-cell and spatial lipidomics via imaging mass spectrometry. Such mapping will define the microenvironments driving follicular arrest in PCOS. Complementing this approach, dynamic metabolic tracing of isotope-labeled LCFAs combined with real-time flux analysis will quantify tissue-specific uptake kinetics across PCOS subtypes. Critically, future studies should establish quantitative relationships and metabolic fluxes of fatty acids between key compartments: serum, FF, and ovarian cells (granulosa and theca), and elucidate their connections to adipose tissue lipid reservoirs. Understanding this multi-compartment metabolic crosstalk is essential to decipher how systemic metabolic signals are relayed to the ovary, ultimately influencing follicular development and ovarian function in PCOS.
2. Deconvoluting Receptor Signaling Networks: validate the FABP5-PI3K/AKT axis in granulosa cell proliferation using organoid models. Determine how CD36 palmitoylation fine-tunes MUFA/SFA selectivity in PCOS adipocytes. Investigate the FFAR4-PPAR*γ* co-activation as a synergistic target for ameliorating IR and hyperandrogenism.
3. Bridging the Translational Gap: develop multi-omics signatures integrating serum LCFA ratios (e.g., n-6/n-3), soluble CD36 (sCD36), and FABP4 levels to stratify PCOS subtypes. Test n-3 PUFA + FFAR4 agonist combotherapy in HA-PCOS animal models. Explore SCD1 inhibitors for normalizing MUFA/SFA balance without compromising oocyte quality.
4. Addressing Unanswered Paradoxes: Resolve why MUFAs show both protective (anti-lipotoxic) and detrimental (pro-inflammatory) effects using tissue-specific knockout models.
5. Integrating Systems Medicine Approaches: An intriguing, yet to be fully tested, hypothesis is that microbiota-derived LCFAs (such as myristoleic acid, MOA) could serve as key mediators linking gut dysbiosis to ovarian inflammation in PCOS. However, direct causal evidence in PCOS, requiring interventional studies in PCOS animal models (e.g., via fecal microbiota transplantation or specific metabolite administration), is currently lacking and represents a critical avenue for future research.
6. Establishing Causal Links from Correlative Observations: Many associations between specific LCFA profiles and PCOS phenotypes summarized herein are derived from clinical correlation studies. A critical future step is to employ genetically engineered PCOS animal models (e.g., tissue-specific knockout/overexpression of targets like FABP5, CD36) and isotopic metabolic tracing to move beyond correlation and definitively establish the causal roles of these lipid pathways in driving IR, hyperandrogenism, and follicular arrest.

## Glossary

AA: Arachidonic Acid
ACC: Acetyl-CoA Carboxylase
ACS: Acyl-CoA Synthetase
AGEs: Advanced Glycation End-products
A-FABP: Adipocyte Fatty Acid-Binding Protein (FABP4)
ALA: *α*-Linolenic Acid
AMPK: AMP-activated protein kinase
BA: Behenic Acid
BMI: Body Mass Index
DGLA: Dihomo-γ-linolenic Acid
DHA: Docosahexaenoic Acid
DHT: Dihydrotestosterone
DPA: Docosapentaenoic acid
ECM: Extracellular Matrix
EPA: Eicosapentaenoic Acid
ER: Endoplasmic Reticulum
FABPs: Fatty Acid-Binding Proteins
FADS: Fatty acid desaturases
FAO: Fatty Acid Oxidation
FASN: Fatty Acid Synthase
FATPs: Fatty Acid Transport Proteins
FF: Follicular Fluid
FFAR1: Free Fatty Acid Receptor 1 (GPR40)
FFAR4: Free Fatty Acid Receptor 4 (GPR120)
FSHR: Follicle-Stimulating Hormone Receptor
GCs: Granulosa Cells
GLA: γ-Linolenic Acid
GLP-1: Glucagon-Like Peptide-1
GPCRs: G Protein-Coupled Receptors
GWAS: Genome-wide association study
HA: Hyperandrogenism
HDL-C: High-Density Lipoprotein Cholesterol-C
HOMA-IR: Homeostatic Model Assessment of Insulin Resistance
HPO: Hypothalamic–Pituitary–Ovarian
IR: Insulin Resistance
KGN: Human Ovarian Granulosa Tumor Cell Line
LA: Linoleic Acid
LCFAs: Long-Chain Fatty Acids
LC-SFAs: Longer Chain Saturated Fatty Acids
LDL-C: Low-Density Lipoprotein Cholesterol-C
MA: Myristic Acid
MCFA: Medium-Chain Fatty Acid
MOA: Myristoleic Acid
MUFAs: Monounsaturated Fatty Acids
NF-κB: Nuclear Factor kappa-light-chain-enhancer of activated B cells
non-HDL-C: Non-High-Density Lipoprotein Cholesterol-C
OA: Oleic Acid
OD: Oligomenorrhea or Anovulation
OS: Oxidative Stress
PA: Palmitic Acid
PCOM: Polycystic Ovarian Morphology
PCOS: Polycystic Ovary Syndrome
PGC1a: Peroxisome proliferator-activated receptor gamma coactivator 1-alpha
PI3K: Phosphoinositide 3-Kinase
POA: Palmitoleic Acid
PPARs: Peroxisome Proliferator-Activated Receptors
PUFAs: Polyunsaturated Fatty Acids
ROS: Reactive Oxygen Species
SA: Stearic acid
SCD1: Stearoyl-CoA Desaturase 1
SFAs: Saturated Fatty Acids
SHBG: Sex Hormone-Binding Globulin
SNP: Single Nucleotide Polymorphism
sCD36: Soluble CD36
SIRT1: Sirtuin 1
SLC27: Solute Carrier Family 27
TLRs: Toll-Like Receptors
UFAs: Unsaturated Fatty Acids
VLCFA: Very Long-Chain Fatty Acid
WAT: White Adipose Tissue
Wnt: Wingless/Integrated Signal Pathway
